# Mr. Welsh's Case of Inverted Uterus

**Published:** 1801-05

**Authors:** George Welsh

**Affiliations:** Chelmsford


### *To the Editors of the Medical and Phyfical Journal.*

Gentlemen,

If the following Cafe fhould be deemed fufficiently interefting to merit infertion in the Medical Journal, as giving a concife ftatement of a difeafe not very frequently occurring in private practice, or terminating favorably, I fhall feel myfelf happy in having communicated it. I am, &c.

*Chelmsford, Feb.* 12, 1801. GEORGE WELSH.

---

In July laft, I was requefted to attend a young married lady of delicate frame and habit, and who was far advanced in her her pregnancy of a fecond child. Shortly after, I was fent to, and informed fhe had been unwell fome hours, with trifling pains, which her friends were apprehenfive would terminate in labour. Upon my arrival, I found the os tincæ open, and the head of the child low down, preceded by the membranes, which protruded in the form of a fmall bag, diftended by the liquor amnii. As her pains had not been ftrong, but recurred at fhort intervasl, the os uteri had dilated gradually, and the labour advanced flowly; but by rupturing the membranes, the waters were evacuated, and her pains growing ftronger, fhe was, in about two hours after, delivered. On dividing the funis to remove the child, I was extremely furprifed to find the uterus completely inverted, and laying without the labia, on the thigh, with the placenta firmly adhering to its fundus; and my patient low and weak, with ficknefs and frequent fainting fits. I could not but feel fenfibly the danger of her fituation, of which I apprized her friends, and expreffed a wifh, that a friend of mine in the town, (Mr. Bird) who defervedly poffeffes the higheft character for profeffional abilities, might be alfo employed; but as fhe declined any other affiftance, I endeavoured to feparate the placenta, which was foon effected, and as the veffels did not bleed much, the uterus was returned without any very confiderable hæmorrhage; but as her fainting fits and ficknefs continued with a low and weak pulfe, which was at times almoft imperceptible, I directed a cordial draught to be given, with Tinct. Opii et Liquor Vol. Cornu cervi, aa. gts. xx. and in the courfe of a very few hours had the fatisfaction to find her tolerably eafy, more cheerful, and her pulfe confiderably ftronger. On the following morning I faw her, and found fhe had paffed a quiet night, though without fleep; had fuffered but little from the after-pains, and was free from fever, but with great languor and debility: her ficknefs and faintings had not recurred. I directed a continuance of cordial medicines. The next day fhe was confiderably amended, and eafier; had flept during the night; had paffed urine without pain, and did not complain of uneafinefs in the parts. Her pulfe was much ftronger; and the uterine difcharge not more copious than is ufual; nor did fhe experience a much greater degree of inconvenience than generally refults from labour to delicate women; but as fhe had not had ftools, I directed an enema to de adminiftered, which had the wifhed effect.

From this time fhe continued rapidly to mend; and by taking the bark in as light a form as her ftomach would bear, was foon enabled to ufe exercife, and reftored to her former ftate of health.

On enquiry, I learned the inverfion was to be attributed to a naturally lax habit, and the placenta having been too forcibly extracted at a former period,—a circumftance I was not acquainted with till fome days after her delivery.

I have the pleafure to add, my patient enjoys good health at prefent, and feels no particular inconvenience from an accident fo alarming, and to which fhe will moft probably be hereafter fubject on thofe occafions.

